# Patient Perceptions on the Role of Informal Caregiver Support in Managing Advanced COPD

**DOI:** 10.1177/23743735251341727

**Published:** 2025-07-23

**Authors:** Barbara Gonçalves, Eileen Harkess-Murphy, Audrey Cund, Caroline Sime, Joanne Lusher

**Affiliations:** 1 School of Health and Life Sciences, University of the West of Scotland, Paisley, UK; 2 Scottish Partnership for Palliative Care, Edinburgh, UK; 3 Provost's Group, 11624 Regent's University London, London, UK

**Keywords:** COPD, palliative care, informal caregivers, social isolation, family relations

## Abstract

People with advanced chronic obstructive pulmonary disease (COPD) experience a high physical burden which limits their activities and leads to social isolation, loneliness, and burdening social networks, with informal caregivers playing a crucial role in managing their condition. This study explored the views of people with COPD on informal caregiving, highlighting lifestyle adjustments and caregiver–patient dynamics. A qualitative study using semistructured interviews was conducted with 22 participants with advanced COPD recruited from palliative care services. Three themes emerged: patient autonomy, adapting to life under medical constraints and caregiver's understanding of patients’ needs. Health deterioration caused patient dependency on caregivers, along with feelings of burden, frustration, and distress. Good information provision and education improve caregiver involvement and patients’ self-management. Participants without informed caregiver support struggled with anxiety and disease management. Caregiver–patient relationship quality impacted the caregiver's involvement. In conclusion, patient dependency in advanced COPD leads to frustration and distress, highlighting the need for caregiver education and support. Effective information provision and early involvement of caregivers is essential to improving care quality, reducing distress, and supporting holistic palliative care.

## Introduction

Chronic obstructive pulmonary disease (COPD) is a preventable and treatable disease, characterized by progressive and not fully reversible airflow limitation due to airway or/and alveoli abnormalities, typically caused by exposure to noxious gases or particles.^1^ An estimated 391 million people are living with COPD and it ranks as the third leading cause of death worldwide.^2,3^ Given its prevalence and impact, it should become a public health priority.^4^

People with COPD and their informal caregivers face life with various limitations. COPD-related respiratory impairments cause considerable distressing symptoms, functional limitations, reduced self-care ability, increased anxiety, panic attacks, and depression, with physical symptoms often aggravating psychological symptoms and vice versa, contributing to developing a vicious circle.^5-7^ COPD-related oxygen depletion often necessitates oxygen therapy, which can ease symptoms and enhance mobility but may also lead to dependance, restricting individuals to their homes or making them reliant on portable oxygen for outings.^8,9^ Moreover, for some patients, the fact of having oxygen therapy made the disease visible, which was associated with stigma, possibly leading to their social isolation.^10^ Experiencing physical symptoms forces individuals to reprioritize their lives and stop previous activities such as jobs and home chores, increasing dependance on caregivers and assistive devices while leading to social isolation, loneliness, existential suffering, and loss of freedom.^9,11^ As a consequence, their feelings of burden to family are relatively common, and the reluctance to rely on others can make the inability to perform everyday tasks especially frustrating and disheartening.^8,9^ Informal caregivers, however, often feel they have little choice in assuming the caregiving role, describing it as an inevitable process of gradually taking on more responsibility as the patient's functional abilities decline over the long course of the disease.^12^ This dynamic nature of caregiving underscores the importance of understanding how the quality of the relationship between caregivers and care recipients influences the overall quality of care.^13^

The progressive nature of COPD, marked by long phases of health decline and acute exacerbations, shifts care from life-prolonging therapies to palliative interventions emphasizing holistic support, including emotional, social, and practical assistance.^14,15^ Moreover, severe exacerbations of COPD can be challenging to manage and often surpass the capabilities of community teams,^16^ highlighting the need for early palliative care integration. The potential benefits of early integration of palliative care for people with COPD include improved disease acceptance, psychosocial support, and symptom management. People with COPD receiving palliative care, including psychological and educational interventions often feel confident about managing their disease.^17^ Informal caregivers play a vital role in palliative care, ensuring holistic support while managing care needs and coping with emotional strain as COPD progresses.^18,19^ Just as individuals with COPD, caregivers also benefit from the availability of direct access to healthcare professionals, which provides essential support during patient decline.^20^

Although research has examined caregiver burden in COPD, there remains a limited understanding of people with COPD's perspective on caregiver roles, particularly in disease management and palliative care, remains limited. Most studies focus on caregivers’ experiences rather than the views of individuals with COPD on the support they receive. This study aimed to explore how people with advanced COPD perceive their needs and the role of informal caregivers in supporting them, particularly in managing oxygen dependency and the challenges associated with lifestyle restrictions imposed by the disease.

## Methods

### Study Design

A qualitative exploratory descriptive design was employed, offering an objective and in-depth exploration of participants’ experiences and challenges in disease management.^21^ This approach allowed us to capture the complexities of COPD caregiving dynamics, providing a clear and authentic representation of participants’ perspectives. This study adhered to the COREQ guideline for reporting qualitative research.^22^

### Ethical Approval and Consent to Participate

Ethical approval was obtained from London Stanmore NHS Ethics Committee (reference19/LO/255972). All participants provided written informed consent and received a debrief sheet and a privacy statement.

### Participants and Procedures

This research was conducted in Scotland. To minimize selection bias, participants were recruited using purposive sampling across multiple sites. Independent healthcare staff screened participants and provided study information in person or via phone. Participants with doctor-diagnosed advanced COPD (Global Initiative for Chronic Obstructive Lung Disease stage II–IV) were recruited from one Breathe Easy clinic offering disease-specific support and 3 day hospices providing general support or rehabilitation programs. Eligibility required attending services for at least 2 visits or, for COPD stage II, receiving services for several years. Exclusion criteria included psychiatric or cognitive disorders, other respiratory or progressive diseases, and active cancer.

### Data Collection

Semistructured interviews were conducted by the first author, an independent healthcare professional. Written consent was obtained before interviews, which took place in participants’ homes, healthcare facilities and a workplace, with 2 participants accompanied by spouses. Interviews lasted 25 to 105 min (average 42) and were guided by open-ended questions exploring daily experiences, relationships, and patient needs. Topics included: (1) COPD's impact on daily life, (2) seeking or receiving support, and (3) desired caregiver support. Field notes were collected, and recruitment continued until data saturation. All interviews were audiorecorded, transcribed, and anonymized.

### Data Analysis

Interviews were analyzed using Framework Analysis, a systematic method ensuring transparency and structured thematic development through familiarization, coding, charting, and interpretation.^23^ The first author was responsible for transcription, coding and theme development using NVivo12 software, while coauthors reviewed the framework and provided feedback at each stage of the process. To minimize bias, the analysis was conducted collaboratively, with all coauthors actively participating in reviewing and discussing analysis, including the development of themes and the creation of the matrix chart through discussions and feedback loops. An audit trail documented coding decisions for transparency. No interview was repeated and participants did not provide feedback on transcripts or findings. Findings were further refined through peer debriefing with palliative care researchers and presented at conferences.

### Participant Characteristics

The study included 22 participants. Of the 26 people with advanced COPD at the Breathe Easy clinic, 6 declined to participate, 2 had lung infections, 1 was unreachable, and 1 passed away. Of 11 eligible patients from 5 hospices, 6 participated. All participants were White, with a mean age of 67.23 ± 8.67 years and Forced Expiratory Volume in 1 second predicted (FEV1 pred.) of 36.92% ± 13.79%. Participant characteristics are displayed in Table 1.

**Table 1.** Participants Characteristics.

No	Gender	FEV1% pred	Age	Marital status	Employment status	Oxygen therapy	Informal support	Living situation
1	Female	N/D	79	Widowed	Retired	Yes	Children	Alone
2	Male	N/D	85	Widowed	Retired	Yes	No one	Care home
3	Female	27	63	Married	Unemployed	No	Children Grandchildren Friends Neighbors	Cohabiting
4	Female	34	45	Single	Unemployed	Yes	Mother	Cohabiting
5	Male	26	67	Partnership	Retired	Yes	Partner	Cohabiting
6	Female	21	62	Married	Retired	Yes	Spouse Children Friends	Cohabiting
7	Male	44	78	Widowed	Retired	Yes	Children Grandchildren	Alone
8	Female	20	66	Divorced	Retired	Yes	Children Grandchildren Friends	Alone
9	Male	41	68	Partnership	Retired	No	Partner Children	Cohabiting
10	Female	33	63	Divorced	Retired	Yes	Cousins Grandchildren	Alone
11	Female	51	64	Divorced	Unemployed	No	Children Grandchildren	Cohabiting
12	Male	27	73	Married	Retired	No	Spouse Children Grandchildren Friends	Cohabiting
13	Female	33	61	Partnership	Retired	No	Partner Children Friends	Cohabiting
14	Male	21	54	Single	Employed	Yes	Children Friends	Cohabiting 3 days/week
15	Female	45	73	Married	Retired	Yes	Spouse	Cohabiting
16	Female	43	64	Married	Retired	No	Spouse	Cohabiting
17	Female	37	66	Married	Retired	Yes	Spouse Children	Cohabiting
18	Female	72	70	Divorced	Retired	No	Children Neighbors	Alone
19	Male	35	62	Married	Retired	Yes	Spouse	Cohabiting
20	Male	28	75	Widowed	Retired	No	Children	Assisted living
21	Male	65	67	Married	Retired	No	Spouse	Cohabiting
22	Male	36	74	Divorced	Retired	No	Children Cousins Grandchildren Friends	Alone

## Findings

Three themes identified were: (1) Patient autonomy, (2) Adapting to life under medical constraints, and (3) Caregivers’ understanding of patients’ needs (see Table 2).

**Table 2.** Themes and Subthemes.

Theme	Subthemes	Illustrative quote
Patient autonomy	Caregiver–patient dependency	*I think it's more or less just getting out and about and being able to do the things I used to be able to do without having to carry an oxygen tank on my back. Think that's the most important thing.* (P19) *If my wife didn’t have a car, I wouldn’t get out.* (P09) *As much as the doctor says, “There’re hundreds of people like you!” I never see them. I never see them walking about with one of these things, never. And I don’t know why I never see them, maybe I don’t go out enough. In places I go to, I don’t see them. So, that puts me off a wee bit.* (P08)
	Guilt and the emotional burden	*I don’t want my children to say, “What can I do for you, Dad? Tell me to do this, can I do this for you?” (…) At the moment, I don’t want my children to be involved. I don’t think it’d be fair on them at the moment. But if and when it does get worse, which it will, I think then, having them here could help them better understand what's happening.* (P14)
	Caregiver sacrifices and relationship dynamics	*My daughter and my son can take me out when it's possible. It's not very often, because they’ve got their own jobs and their own kids to look after. I try to be still independent rather than depending on somebody. They say: “Right, we’ll take you somewhere and you can go to that,” like a night out but I say no. (I feel) I’m putting them under … not pressure, but obligement sort of thing. And I don’t want it….* (P9)
Adapting to life under medical constraints	Dependency on oxygen therapy and adjusting routines	*I can’t do anything. If I need to go anywhere, I need to wear oxygen. I don’t want anybody to see me wearing oxygen. That's why I wanted the wee-er tanks because you can put them into a duffle bag, but the big ones you had to carry on your back. I’ve not been able to go out because the oxygen bottles were too big. They’re too heavy to carry. They’ve given me smaller ones which is a bit of a help.* (P19) *It's annoying that you cannot get out and do things. Very frustrating. Simple wee jobs that you used to do, it's an operation trying to get things done. I’m very slow in getting things done. But the body just won’t take it, and you’ve just got to sit and wait until oxygen levels catch up with you.* (P07)
	Social and activity limitations	*My son walks the dog. That's a big thing as well for me, because I thought I’d get out more walking the dog more. I can’t walk, so I can’t walk the dog either, so that's why it's good that my boy does it.* (P14) *My social life is basically visiting and seeing family. I used to do voluntary work, voluntary gardening. But I’ve not been able to do that since last year. (…) And no, my social life is really confined to family at the minute*. (P11)
	Lifestyle adjustments	*I’m limited to the amount of oxygen that the tanks hold. I used to be a huge concertgoer. I don’t do that anymore, because it's a cylinder that only lasts me about an hour. So, it's been a real struggle.* (P14)
Caregivers’ understanding of patients’ needs	Impact of caregiver education	*I feel if people understand how I’m feeling, I feel more supported. My family and my people, that come here do understand and they’re very kind. And I think that's what you need, somebody that realizes that they have to give you a shower.* (P8) *She (wife) knows what to do with me if I go into a panic. For my breathing, slow down, shoulders down, slow down. And then she talks me into it. Talks me down.* (P12)
	Shift in social roles	*The lassie here got me a befriender. Of course, the husband wasn’t happy with that. “Happy to look after you,” he wasn’t happy about it in the beginning because “that's what I’m here for, to take you out.” I said it's just I need a break away from you the same as you’re needing a bigger break away from me that's why I go to the hospice.* (P17) *That's a horrible feeling to be dependent on everybody. And my husband, I don’t know what I’d do without him. I really don’t. He's not the most patient of men, you know, he's one of these … maybe not used to doing housework. If I say to him, “The place needs hoovering,” the atmosphere starts changing and things like that, and I try not to annoy him.* (P15)
	Limited understanding and loss of support networks	*They know I’ve got COPD, and they know I’m on oxygen. I don’t get embarrassed in front of my family but they don’t understand like I understand the cause of COPD and what COPD is. It's a lung disease which is never going to go away anyway. So, I try and sit and explain that and I feel as if they’re not taking that in.* (P19) *You just have to meet the right person that you can trust, who you can talk to, like a very close friend. And I lost all of them.* (P16)

### Patient Autonomy

Maintaining autonomy was crucial for upholding a good mood and limiting psychological distress. The ability to manage themselves was, for some, the most substantial aspect of life as disabilities caused by the disease forced them to ask caregivers for help, which distressed them. However, informal support was important as patients could not leave home without the carers’ help.

Health deterioration led to dependency on caregivers, causing a feeling of being a burden. Patients were concerned that the caregivers had to make significant lifestyle sacrifices to help them. Nearly all participants’ partners assumed caregiving roles, which sometimes affected spousal relationships. Participants living with children or occasionally receiving their help also expressed guilt for depending on them, feeling they should be the ones providing support instead. Some participants noted that they would not want their children to be involved in their care.

Although some participants learnt how to live with this disease, being unable to perform previous activities or being slow when doing them led to frustration and annoyance, often needing caregivers to step in and help manage day-to-day tasks. Participants felt upset because they had not encountered other patients with COPD and were not familiar with their experiences. Participants were recruited from palliative care services that offered support groups and rehabilitation programs, providing additional resources beyond standard care. However, some refused to attend them due to distance, unwilling to inconvenience their caregivers with transportation and logistics or to sacrifice their time.

### Adapting to Life Under Medical Constraints

Living with advanced COPD often entailed polypharmacy, with a diverse effect on the patients. As the disease progressed, patients increasingly required support such as oxygen therapy, which significantly affected their daily lives. It was connected with limited mobility, reduced social contact, and embarrassment. Physical restrictions and increased oxygen dependance forced many participants to forgo activities like dog walking, strolling, or shopping, leaving them reliant on their caregivers for support. Also, the use of oxygen negatively impacted the participants’ ability to remain active and socialize effectively. Some participants described being housebound as “becoming a shut-in” and “stuck in prison,” and their social contact was limited to their families and friends, with caregivers often being their primary source of interaction. Also, leaving home with mobile oxygen depended on its battery life and made leaving home challenging.

### Caregivers’ Understanding of Patients’ Needs

The analysis of support from family or other informal caregivers revealed a clear connection: the more the caregivers were educated about COPD and involved in their care, the more sensitive they became to patients’ needs. Participants whose caregivers understood COPD felt supported by them and were more confident, as they knew the treatment as well as their needs. Likewise, having a caregiver involved in care since the diagnosis was made, contributed to patients feeling supported as caregivers helped them to be active within their capacity. Receiving help to go out was advantageous in maintaining their lifestyle, and having their needs listened to contributed to their overall satisfaction with care and their sense of being well supported. When a caregiver understood the patients’ needs, they seemed more interested in learning about the disease and its treatments. Educated caregivers were particularly helpful in managing panic attacks caused by breathlessness through effective breathing control techniques.

Close relationships with caregivers, whether established before or after diagnosis, impacted patients’ satisfaction with informal caregiving. Supportive and involved caregivers were appreciated for assuming responsibility for patient care, often at the expense of their own hobbies and free time. Concerns about caregivers’ mental well-being arose among some participants due to this sacrifice, prompting suggestions for them to seek respite through befriender services or daycare programs. In some cases, social roles shifted, with husbands taking over household responsibilities typically performed by wives, such as cleaning. This adjustment sometimes strained family dynamics as wives felt they were burdening their husbands.

Having a caregiver at home did not necessarily mean feeling supported. Notably, patients with caregivers who understood the diagnosis or who were sensitive to their needs were less frustrated. Participants whose caregivers did not understand their diagnosis reported they felt lonely, frustrated, and not supported, regardless of the severity of the disease. Despite living with a spouse and having regular support from children, some patients still did not feel understood as families knew little about COPD and need for oxygen. In advanced stages of COPD, it was deemed too late to engage spouses more actively in the care process, a step that should have been taken earlier in the diagnosis. Had educational programs been introduced to them from the outset, they might have been able to contribute to caregivers becoming more involved and supportive. Nonetheless, participants emphasized the importance of empathetic attitudes from informal caregivers, making them feel understood and less isolated with their disease burden, which in turn, had a positive impact on their quality of life.

## Discussion

This study examined how people with advanced COPD perceive their needs within the context of palliative care, highlighting both psychological and social aspects and the pivotal role of informal caregivers in disease management and emotional support. The findings presented offer specific strategies to enhance caregiver potential involvement tailored to the challenges of COPD management in palliative care settings. The analysis revealed the negative effect of losing independence caused by changes in lifestyle due to disease progression and the importance of the caregiver's understanding and involvement in care. While informal caregivers have a vital role, the caregiving process itself is often challenging and multifaceted. In contrast to previous studies which offer mixed opinions on caregiver support,^24^ our study highlights that recipients highly appreciated caregivers’ participation and involvement in additional interventions, primarily because being well-informed about the disease enabled caregivers to provide empathetic support tailored to the patients’ needs. This finding is consistent with previous research which accentuates the helpfulness of family networks while living with a severe and chronic condition, as social support is a protective factor for the well-being of patients.^25^ In line with the results of this study, emotional support and caregivers’ help with everyday tasks can contribute to patients’ adjustment to the new life situation when living with a chronic condition.^26^ Furthermore, patient-tailored self-management interventions with facilitated social support are advised for people with COPD, as they may introduce opportunities to positively impact self-management behaviors.^27^ While our findings build on this recommendation, they also introduce a novel perspective by illustrating how caregivers’ understanding and involvement can notably enhance patients’ self-management and well-being. This accentuates the importance of caregiver participation in care and the potential benefits of tailored interventions aimed at improving patient outcomes and self-management behaviors.

The present study indicates that having a close relationship with a caregiver prior to or after diagnosis allowed patients to be more open about their needs. Dependance often strained spousal relationships and heightened reliance on children for support, revealing the emotional and practical complexities of COPD caregiving. Essentially, when individuals with COPD did not have a close relationship with their caregivers, it was more difficult to involve them in the care and expect them to assist in everyday activities. This preexisting closeness enabled caregivers to provide more effective support, leading to improved outcomes such as reduced anxiety and enhanced self-management. Other authors found additional positive contributions from caregivers. The absence of a caregiver has been linked to an increased need for palliative care.^28^ Also, Scheerens et al^29^, observed that involving informal caregivers can enhance early integrated palliative care. Furthermore, Aasbø et al^30^, concluded that caregivers could have a crucial role in the improvement of patients’ self-care, which is corroborated by the views of the participants from the present study. Both studies observed that caregivers contributed to patients’ maintaining disease stability, as well as to preventing their emotional distress and breathlessness, especially through education about COPD. Caregivers helped manage dyspnea by maintaining clean homes, learning breathing techniques, and assisting with activities that could induce breathlessness. Likewise, they were instrumental in preventing emotional distress by alleviating feelings of burden, managing triggers, and providing joy and purpose in patients’ lives. Informal caregivers could contribute to unburdening patients by contacting healthcare professionals on their behalf,^30,31^ when patients were sometimes unsure of their need for consultation or did not recognize that the deterioration of their condition required a physician's opinion.^32^ Finally, they played a significant role in motivating patients to take their medications properly and in monitoring disease exacerbation.^33,34^ Our findings showed that caregivers involvement has the potential to improve patients’ health outcomes and self-management skills, especially when integrating them into care during the early stages of the disease rather than in the palliative care stages. Therefore, the importance of involving informal caregivers in initial consultations with healthcare professionals is stressed here to foster an understanding of the disease trajectory and improving their collaboration with healthcare providers.

Further, there is an ambiguous relationship between being at risk of mental health problems or experiencing psychological distress and having a social network or living alone. Previous research showed that the prevalence of common mental disorders was higher in people who live alone.^35^ Correspondingly, considering the physical burden of people with COPD, which already puts them at a higher risk of developing mental health disorders, living alone is another factor which may contribute to developing depression, even in those in earlier stages of the disease.^36^ Among those with advanced COPD living with family, 18.3% reported inadequate social support, which was associated with worse clinical outcomes, including increased symptom severity, symptoms of depression, and higher levels of care dependency.^37^ The results of the present study indicated that the presence of a caregiver at home did not consistently result in patients feeling adequately supported. Instead, we suggest that this feeling seemed to be closely associated with the caregivers’ understanding of COPD, highlighting the key role of early education and sufficient information provision in enhancing caregiver involvement and improving patient well-being. Therefore, in line with previous research we emphasize the importance of caregiver's partaking in care and the quality of caregiver relationships in COPD patients’ perceived support, and advocate for informal caregivers’ participation in support groups or educational interventions to enhance patient self-management.^38-40^

## Limitations and Directions for Further Research

Participants were recruited from a limited pool of palliative care services; thus, the full spectrum of patients, including those not referred or doctor-diagnosed, has not been considered. Future research could explore the dynamics of caregiving relationships and their impact on care quality.

## Conclusion

The informal caregivers’ role is vital in developing proper self-management by boosting patients’ confidence and decreasing psychological distress. When caregivers comprehend the underlying causes of patients’ feelings and behavior, then the patients may experience less psychological distress as they feel their needs are being met and supported. Early education of caregivers is undeniably crucial to acquiring an understanding of the diagnosis and, subsequently, it fosters the involvement of caregivers in patient care. Consequently, patients experience less psychological burden when they feel well taken care of and understood. Therefore, the role of caregivers appears to be critical in maintaining and improving patients’ self-management and, as such, involvement in the care of patients should be encouraged and facilitated. It should receive more attention, especially when introducing caregivers into the care of patients from the early stages of diagnosis, considering the chronic nature of COPD and the potential for more effective self-management if introduced early.

## Supplemental Material

sj-docx-1-jpx-10.1177_23743735251341727 - Supplemental material for Patient Perceptions on the Role of Informal Caregiver Support in Managing Advanced COPD Supplemental material, sj-docx-1-jpx-10.1177_23743735251341727 for Patient Perceptions on the Role of Informal Caregiver Support in Managing Advanced COPD by Barbara Gonçalves, Eileen Harkess-Murphy, Audrey Cund, Caroline Sime and Joanne Lusher in Journal of Patient Experience
