# Supplementary material for: Patient Perceptions on the Role of Informal Caregiver Support in Managing Advanced COPD
Source: J Patient Exp. 2025 Jul 23;12:23743735251341727. doi: 10.1177/23743735251341727 (PMC12301613; doi:10.1177/23743735251341727)
Supplement: sj-docx-1-jpx-10.1177_23743735251341727 - Supplemental material for Patient Perceptions on the Role of Informal Caregiver Support in Managing Advanced COPD [file sj-docx-1-jpx-10.1177_23743735251341727.docx]

## **Supplementary File 1. COREQ (Consolidated criteria for Reporting Qualitative research) Checklist**

|  | **Item no.** | Guide Question/Description | Reported on page no. |
| --- | --- | --- | --- |
| **Domain 1: Research team and reflexivity** | | | |
| *Personal characteristics* | | |  |
| Interviewer/facilitator | 1 | Which author/s conducted the interview or focus group? | 5 |
| Credentials | 2 | What were the researcher’s credentials? E.g. PhD, MD | Title page |
| Occupation | 3 | What was their occupation at the time of the study? | 5 |
| Gender | 4 | Was the researcher male or female? | Title page |
| Experience and training | 5 | What experience or training did the researcher have? | 5 |
| *Relationship with participants* | | |  |
| Relationship established | 6 | Was a relationship established prior to study commencement | 5 |
| Participants knowledge of the interviewer | 7 | What did the participants know about the researcher? E.g. personal goals, reasons for doing the research | 4 |
| Interviewer characteristics | 8 | What characteristics were reported about the interviewer/facilitator? E.g. Bias, assumptions, reasons and interests in the research topic | 5 |
| **Domain 2: Study design** | | | |
| *Theoretical framework* | | |  |
| Methodological orientation and theory | 9 | What methodological orientation was stated to underpin the study? E.g. grounded theory, discourse analysis, ethnography, phenomenology, content analysis | 4 |
| *Participants selection* | | |  |
| Sampling | 10 | How were participants selected? E.g. purposive, convenience, consecutive, snowball | 4 |
| Method of approach | 11 | How were participants approached? E.g. face-to-face, telephone, mail, email | 4 |
| Sample size | 12 | How many participants were in the study? | 6 |
| Non-participation | 13 | How many people refused to participate or dropped out? Reasons? | 6 |
| *Setting* |  |  |  |
| Setting of data collection | 14 | Where was the data collected? E.g. home, clinic, workplace | 5 |
| Presence of non-participants | 15 | Was anyone else present besides the participants and researchers? | 5 |
| Description of sample | 16 | What are important characteristics of the sample? E.g. demographic data, date | 6-7 |
| *Data collection* | | |  |
| Interview guide | 17 | Were questions, prompts, guides provided by the authors? Was it pilot tested? | 5 |
| Repeat interviews | 18 | Were repeat interviews carried out? If yes, how many? | 5 |
| Audio/visual recording | 19 | Did the research use audio or visual recording to collect the data? | 5 |
| Field notes | 20 | Were field notes made during and/or after the interview or focus group? | 5 |
| Duration | 21 | What was the duration of the interviews or focus groups? | 5 |
| Data saturation | 22 | Was data saturation discussed? | 5 |
| Transcripts returned | 23 | Were transcripts returned to participants for comment and/or correction? | 5 |
| **Domain 3: analysis and findings** | | | |
| *Data analysis* | | |  |
| Number of data coders | 24 | How many data coders coded the data? | 5 |
| Description of the coding tree | 25 | Did authors provide a description of the coding tree? | 5 |
| Derivation of themes | 26 | Were themes identified in advance or derived from the data? | 5 |
| Software | 27 | What software, if applicable, was used to manage the data? | 5 |
| Participant checking | 28 | Did participants provide feedback on the findings? | 5 |
| *Reporting* |  |  |  |
| Quotations presented | 29 | Were participant quotations presented to illustrate the themes/findings? Was each question identified? E.g. participant number | 7-10 |
| Data and findings consistent | 30 | Was there consistency between the data presented and the findings? | 7-13 |
| Clarity of major themes | 31 | Were major themes clearly presented in the findings? | 7-13 |
| Clarity of minor themes | 32 | Is there a description of diverse cases or discussion of minor themes? | 7-10 |
